# Tobacco-Related Knowledge Among Employees at Substance Use Treatment and Medical Healthcare Centers Serving Rural and Medically Underserved Patients with Substance Use Disorders in Texas, USA

**DOI:** 10.3390/ijerph22111701

**Published:** 2025-11-11

**Authors:** Jedidiah A. Feyisetan, Maggie Britton, Tzuan A. Chen, Isabel Martinez Leal, Mhyank S. Sekhar, Teresa Williams, Kathleen Casey, Ammar D. Siddiqi, Lorraine R. Reitzel

**Affiliations:** 1 Department of Behavioral Science, University of Texas MD Anderson Cancer Center, 1155 Pressler Street, Houston, TX 77030, USA; jedidiah.feyisetan@ttuhsc.edu (J.A.F.); mbritton@mdanderson.org (M.B.); bleal@mdanderson.org (I.M.L.); mssekhar@mdanderson.org (M.S.S.);; 2 School of Medicine, Texas Tech University Health Sciences Center, 3601 4th St STOP 6238, Lubbock, TX 79430, USA; 3 Department of Psychological, Health, and Learning Sciences, University of Houston, McElhinney Hall, 3623 Cullen Blvd., Houston, TX 77204, USA; tchen3@central.uh.edu; 4 HEALTH Research Institute, University of Houston, 4349 Martin Luther King Blvd., Houston, TX 77204, USA; 5 Integral Care, 1430 Collier Street, Austin, TX 78704, USA; teresa.williams@integralcare.org (T.W.); kathleen.casey@integralcare.org (K.C.); 6 School of Medicine, University of California, San Francisco, 533 Parnassus Ave., San Francisco, CA 94143, USA

**Keywords:** rural, tobacco cessation intervention, smoking, tobacco use, medically underserved, substance use treatment center, medical healthcare centers, tobacco use disparities, low income/low SES, cancer prevention, professional education and training

## Abstract

Background: Tobacco use, and particularly cigarette smoking, is elevated among patients with substance use disorders (SUDs), resulting in health inequities. In rural and medically underserved areas (MUAs), healthcare access is limited; thus, patients with SUDs may receive care in substance use treatment centers (SUTCs) and/or medical healthcare centers (MHCs). Healthcare providers in these settings should know the importance and benefits of quitting tobacco use for patients with SUDs, as this is essential for effective cessation care. This study examined baseline provider knowledge/training receipt and knowledge gains following training in SUTCs and MHCs serving rural and MUAs of Texas, USA. Methods: From 2021 to 2024, 428 providers from 8 SUTCs and 8 MHCs completed an e-survey on tobacco knowledge and past-year training. They then completed 1 to 1.5 h of training. Knowledge gain was assessed via a 10-item test delivered pre- and post-training. Results: Pre-training knowledge and past-year training rates were low across settings; providers at SUTCs were more likely than those at MHCs to know that persons with behavioral disorders like substance misuse are 2 times more likely to smoke than the general USA population. Both groups demonstrated significant knowledge gains from training (SUTCs: 37.41% and MHCs: 45.92% increases; ps < 0.0001). Conclusions: Findings support the need for routine tobacco training in healthcare centers serving rural and MUAs of Texas. Brief educational sessions may help address provider knowledge gaps and, ultimately, strengthen cessation care and reduce tobacco-related disparities in these settings.

## 1. Introduction

Tobacco use, and particularly cigarette smoking, is a major driver of preventable illness and death in the United States, contributing to a range of chronic conditions, including cardiovascular disease, cancer, and respiratory illness [1]. While public health efforts have significantly reduced smoking in the general population, people with substance use disorders (SUDs), who often also have mental health comorbidities, continue to smoke at more than twice the rate of the general population [2]. As a result, they face a compounded burden of morbidity and mortality. For example, studies show that patients with SUDs are often more likely to die from smoking-related illnesses than from their primary substance use condition for which they seek care [3].

People in rural and medically underserved areas (MUAs) who use tobacco face additional barriers that limit their access to tobacco cessation services. These include shortages of healthcare providers, fewer treatment options, and higher rates of poverty and uninsurance, all of which make it harder for people to get help with nicotine dependence [4]. Texas, home to the largest rural population in the United States, reflects these challenges [5]. While the definition of “rural” varies depending on the defining agency and the intended use, multiple health-related agencies (e.g., Texas State Office of Rural Health, Health Resources and Services Administration [HRSA]) state that 70–80% of Texas’ 254 counties are classified as rural, with even more considered partially rural [6,7,8,9]. Additionally, hundreds of areas across the state are considered medically underserved. According to HRSA, these areas experience shortages of primary care providers, either for the general population or for specific groups such as low-income individuals, Medicaid-eligible residents, and unhoused communities [10,11,12]. These areas have disproportionately fewer hospitals and disproportionately high uninsurance rates, with up to 25% of residents having no medical insurance [6]. In these communities, patients with SUDs may receive care at medical healthcare centers (MHCs), substance use treatment centers (SUTCs), or sometimes both, making both types of centers critical points of intervention for patients’ tobacco use.

Medical Healthcare Centers (MHCs) are facilities that provide comprehensive medical and behavioral health services, including primary care, preventive services, and management of chronic conditions. Substance Use Treatment Centers (SUTCs), in contrast, focus specifically on the treatment of substance use disorders and may offer services such as detoxification, counseling, rehabilitation, and medication-assisted treatment [13]. While both center types serve patients who use tobacco, they differ in their primary goals, patient populations, and clinical workflows. These differences likely influence how providers conceptualize and address tobacco use across settings.

Relatively little is known about how much healthcare providers in these rural/MUA settings know about tobacco use and its impact on patients with SUDs, or whether they have received recent training on how to help those patients quit [14]. Various theories and frameworks (e.g., Theory of Planned Behavior, Knowledge–Attitude–Practice Theory, Theoretical Domains Framework, Social Cognitive Theory) suggest that topical knowledge, which can be increased via educational efforts, is a key upstream factor that ultimately affects behavior and behavior change [15,16,17,18]. Prior work conducted in several Texas healthcare centers, including MHCs and SUTCs in metropolitan areas, also supports that providers’ knowledge is a key predictor of whether tobacco cessation treatment is delivered [19]. Furthermore, similar work has demonstrated that even relatively brief training (e.g., 1–2 h) about tobacco can improve provider knowledge and intervention practices in healthcare settings [20,21,22]. However, most of this research has focused on urban centers, and less is known about how training outcomes vary in rural areas or MUAs, where both tobacco use and healthcare barriers are more pronounced [14]. Without a solid understanding of the health risks of tobacco use or the benefits of quitting for patients with SUDs (e.g., benefits to recovery from other substance use), providers may be less likely to bring up the topic or offer appropriate support [23].

Although rural or medically underserved patients may visit and receive care at MHCs or SUTCs for their SUDs, these healthcare center types—by design—serve different purposes. These differences might influence how prepared or willing providers are to deliver tobacco cessation care, despite the fact that best practice guidelines indicate that all treatment providers should be screening for and treating tobacco use in every clinical encounter, regardless of healthcare center type [24]. For example, it may be that providers in MHCs focus more on chronic disease management and thus may be more familiar with and more likely to follow established clinical guidelines, including those for addressing patients’ tobacco use [24]. On the other hand, healthcare encounters in MHCs are often brief, relative to those in behavioral health settings like SUTCs. These time constraints could lead to limited knowledge and a failure to address patients’ tobacco use—especially when it is not the primary reason for the visit. In contrast, providers in SUTCs already possess the behavioral health skills to treat addictions and so may have more knowledge relative to MHC providers on how to address their patients’ nicotine addiction. However, research suggests that tobacco use is not routinely addressed at SUTCs. One reason is the persistent concern among some SUTC providers that addressing tobacco cessation during treatment could jeopardize patients’ sobriety from other drugs, a belief that has been reported despite evidence showing cessation can support long-term recovery [25].

For example, a landmark national study from 2016 reported that 64% of SUTCs screened patients for tobacco use, only 47.4% provided tobacco cessation counseling, and just 20–26% provided other evidence-based interventions like nicotine replacement therapy and non-nicotine cessation medications [26]. A more recent follow-up study from 2023 cited similar patterns [27]. Additionally, tobacco use rates among treatment providers in SUTCs are known to be elevated (i.e., a smoking-permissive culture) relative to both the general population and MHC providers, which may negatively impact their willingness to address it in their patients [28]. Moreover, recent work on barriers to tobacco care provision show some variation by healthcare center type. Specifically, data from MHC employees in Texas indicated 4 key barriers: (1) relative importance of competing diagnoses (66.7%); (2) lack of time (66.7%); (3) patients are not interested (55.6%); and (4) patients do not comply with treatment (55.6%). In SUTCs, however, 8 key barriers were endorsed: (1) lack of training (67.4%); (2) lack of community resources to refer patients (62.5%); (3) lack of patient education material (58.3%); (4) lack of time (58.3%); (5) relative importance of competing diagnoses (55.1%); (6) lack of provider knowledge or confidence (54.2%); (7) lack of provider interest (50.0%); and (8) patients do not comply with treatment (50.0%) [29]. This may suggest that SUTCs are less equipped to address patients’ tobacco use relative to MHCs because they endorse more barriers to its delivery.

These crucial distinctions between MHCs and SUTCs raise important questions about whether providers in one setting are better equipped than those in the other to provide tobacco-related care. This has implications for designing and targeting workplace interventions to build tobacco screening and intervention provision in these settings to better align community healthcare with evidence-based best practice guidelines and, ultimately, reduce tobacco-related disease inequities. To our knowledge, no prior study has directly compared tobacco-related knowledge and training receipt between MHCs and SUTCs serving rural and/or MUAs. Understanding how these settings differ can help tailor interventions to the specific needs of each healthcare center type and strengthen the delivery of tobacco cessation services in high-need community settings.

The current study was designed to address gaps in the literature by assessing differences in tobacco-related knowledge and recent training among providers at MHCs and SUTCs serving rural and/or MUAs of Texas. Moreover, it examined knowledge gained by providers in each setting following participation in a relatively brief tobacco training session. By evaluating and comparing provider knowledge and training exposure in these settings, this study helps identify where additional education is needed and where capacity-building efforts are likely to have the greatest impact in rural healthcare. Investigating these patterns is a key step toward improving tobacco cessation efforts in rural Texas and, more broadly, reducing the disproportionate burden of tobacco-related disease among people with SUDs.

## 2. Materials and Methods

### 2.1. Participants and Procedures

MHCs and SUTCs throughout Texas were solicited via email to participate in a comprehensive tobacco-free workplace program to build their center’s capacity to address tobacco use with evidence-based intervention. Eligibility for inclusion in the program, called Taking Rural Texas Tobacco Free, was based on whether the center was situated within and/or served individuals from rural or MUAs in Texas. Participants were limited to Texas because the work was funded through a grant from the Cancer Prevention & Research Institute of Texas (PP210003). The Taking Rural Texas Tobacco Free program had several evidence-based core components, one of which was training all employees on the harms of tobacco use amongst individuals with substance use and/or mental health (collectively, “behavioral health”) needs and how to screen and treat tobacco use. More information about this comprehensive tobacco-free workplace program can be gathered from previously published works [30,31].

This grant was initially awarded to The University of Houston (Houston, TX, USA) in June of 2021 and later was transferred to the University of Texas MD Anderson Cancer Center (Houston, TX, USA) in early 2023 based on a change of employer for most of the grant team. Consequently, the project was first approved by the Internal Review Board of the University of Houston (with a waiver of documentation of consent) and later approved by the Quality Improvement Assessment Board at The University of Texas MD Anderson Cancer Center.

Participating centers were enrolled on a rolling basis; data were collected from the center leadership (e.g., Chief Executive Officer, Executive Director, etc.) or their designee and center employees from August 2021 through May 2025. Overall, 16 enrolled healthcare centers (8 MHCs and 8 SUTCs) participated in the procedures relevant to this report: pre-implementation data collection, tobacco training presented by health education specialist personnel on the grant, and pre-and post-training knowledge assessments during the cited period. All participants provided informed consent for participation by reading a descriptive cover letter that preceded the survey questions; data collection was anonymous. The only remuneration associated with the procedures reported herein was a USD 10 Amazon gift card for employees who completed the pre-implementation electronic survey administered via Qualtrics. Specifically, those completing the survey were sent to a second survey link, not associated with their pre-implementation survey endorsements, wherein they could leave their contact information for receipt of the e-gift card. Aside from the employees’ selection of their MHC or SUTC affiliation from a drop-down list of enrolled centers and self-identification as a direct service (healthcare) provider or general employee with no patient contact/treatment responsibilities, no other identifying information was collected.

#### Tobacco Trainings

All employees were invited to participate in the tobacco training, which was delivered by health educators who were certified tobacco treatment specialists on the project team using PowerPoint slides. Live training (either virtual or in person, based on centers’ preferences) was conducted, and all employees were encouraged to attend. Multiple training sessions (42 overall) were conducted at each center (range was 1 to 9) to engage as many employees as possible. If centers requested the availability of asynchronous training to complement the live training and to meet the needs of employees who worked non-business hours, they were additionally provided with a recorded version, which was the case for 3 centers. The duration of the live training ranged from 1 (for 3 centers) to 1.5 h (for 13 centers), based on center request; the recorded training was self-paced but used the 1.5 h training content.

The tobacco training content was based on clinical practice guidelines [32] and included coverage of the following: (1) all types of tobacco products, inclusive of electronic cigarettes/vapes; (2) the (relative) harms associated with the use of various tobacco products, with an emphasis on those associated with the most predominant type of tobacco use (cigarette smoking); (3) why people, particularly those with mental health and substance use diagnoses, use tobacco and how this relates to use and health inequities; (4) common myths, including that persons who use tobacco do not want to quit, with supporting data to refute them; (5) the benefits of quitting tobacco use on physical health, mental health, and substance use recovery, with an emphasis on cigarette smoking cessation; (6) brief evidence-based treatment, with an emphasis on the 5A’s (for patients wanting to quit) and the 5R’s (for patients not ready to quit); (7) FDA-approved medications for cessation; (8) how to refer or connect patients to the Texas Tobacco Quitline and the services provided; and (9) other treatment resources (including text- and app-based national programs).

The training differed slightly for MHCs and SUTCs, as it was adapted to reflect differences in the participants and populations by center type. For example, the MHC training covered the association of tobacco use and several health conditions in greater detail than the SUTC training did, whereas the SUTC training covered the association of tobacco use and other substance use recovery in greater detail than the MHC training did. In both, however, we contextualized the disproportionate use of tobacco among groups—such as those with behavioral healthcare needs and those living in rural areas—as a social justice issue [33]. As such, the content specifically included training on norms-related approaches to change tobacco use behaviors and intentionally focused on not increasing/exacerbating the judgment and stigmatization of these patients who already experience stigma for substance use [34]. These approaches included correcting misperceptions regarding tobacco use behaviors and shifting the onus from tobacco users to the tobacco industry that intentionally targets under-resourced and marginalized groups [35].

### 2.2. Measures

#### 2.2.1. Center Demographics

Information about the centers was gathered via an electronic survey administered to each center’s CEO or their informed designee. This survey was administered after the center joined the project and before program implementation. Survey questions included: (1) What county/counties do you serve? (254 Texas counties were listed and respondents could select all that apply); (2) What is the number of unique patients aged 16 years or older your center served last year? (3) What is the total number of patient contacts your center had last year (please estimate this for patients aged 16 years or older); (4) What is the number of employees across locations participating in this project? If certain departments/divisions (e.g., emergency room, inpatient detox) are participating and not others, please only count employees from participating departments/divisions; and (5) How many of your employees have direct patient contact? The latter item was designed to differentiate healthcare providers from other employees. Consequently, the following clarification was also provided: “Employees with the following credentials (but not only these credentials) are typically those who have direct patient contact: NP, LVN, RN, APN, CNA, MA, QMHP, MD, LCDC, LSW, etc. Employees might also have titles like recovery coach, patient navigator, or peer support specialist.”

The number of rural or partially rural counties and MUAs was calculated based on definitions from the HRSA. Accordingly, rural areas are defined as: (1) non-metropolitan counties; (2) outlying metropolitan counties with no population from an urban area of ≥50,000 people; (3) census tracts with rural–urban commuting (RUCA) area codes 4–10 in metropolitan counties; (4) census tracts with ≥400 square miles and population density of ≤35 people per square mile with RUCA codes 2–3 in metropolitan counties; and (5) census tracts with road ruggedness scale 5 and RUCA codes 2–3 that are ≥20 square miles in area in metropolitan counties [8,9]. Rural counties are defined as those in which all census tracts are rural areas. Partially rural counties are those where at least one (but not all) census tract is rural. MUAs included: (1) geographic areas with a shortage of primary care health services (e.g., a whole county, group of neighboring counties, group of census tracts); and (2) geographic areas with a shortage of primary care health services for specific populations (e.g., individuals experiencing homelessness, with low income, who are Medicaid eligible, etc.) [10,11].

The following variables were calculated based on the above: (1) the numbers of unique counties served; (2) the subset of counties served that were rural (fully or partially); (3) the number of counties served with at least 1 MUA; (4) the number of unique patients served annually; (5) the number of total patient visits annually; (6) the number of employees; and (7) the subset of employees who were healthcare providers. For 2 and 3 above, data were generated at a single point in time using HRSA data available at that time (November 2024). [9,12]

#### 2.2.2. Pre-Implementation Tobacco Knowledge and Past-Year Training

After the completion of the leadership survey, a separate survey was administered to all employees at the participating centers. Questions on the survey included those assessing tobacco- or smoking-related knowledge and past-year training exposure, and are reported herein for healthcare providers with direct patient contact.

Tobacco-related knowledge was assessed via agreement with the following fact-based items: “People with mental health and/or non-nicotine substance use disorders: (1) …are approximately twice as likely as the general population to smoke cigarettes”; (2) …are more likely to die from smoking-related illnesses than from their mental and/or non-nicotine substance use disorder”; (3) …who smoke want to quit smoking and are able to quit smoking”; and (4) …who quit smoking may experience concurrent improvements in some mental health symptoms and/or reductions in non-nicotine substance use.”

Past-year training exposure was assessed by agreement with the following items: (1) “In the last 12 months, have you received any education at your center regarding the hazards of smoking?”; and (2) “In the last 12 months, have you received any training at your center regarding the hazards of smoking and benefits of quitting that are specific to individuals with substance use disorders?”

#### 2.2.3. Pre- and Post-Training Tobacco Knowledge Tests

A 10-item multiple-choice and true/false knowledge test was developed to assess knowledge gained by employees from the 1 to 1.5 h tobacco training that was delivered by project staff. The items on the test directly reflected the content of the training and its learning objectives. Consequently, different versions of the pre- and post-training test were used for MHCs and SUTCs. Both tests used the same core 7 questions; 3 additional items were tailored to each center type. Several items queried cigarette smoking specifically, other items referred to “tobacco use” overall, which was inclusive of electronic nicotine delivery systems per the training content. Once the post-test was completed, the employee received feedback (i.e., correct/incorrect) on all items. For any incorrect answers, they were also given a brief explanation clarifying why the correct response was factually accurate.

Some of the shared items on the knowledge tests included: “Which of the following tobacco treatment medications require a prescription?” (response options = (a) nicotine patch, (b) nicotine inhaler [correct answer], (c) nicotine lozenge, (d) nicotine gum, and (e) all of the above); “Which of the following is NOT one of the 5A’s of tobacco cessation brief intervention?” (response options = (a) ask, (b) arrange, (c) assess, or (d) allow [correct answer]); and “Smoking cessation interventions were associated with ___ increased likelihood of long-term alcohol and drug abstinence following substance abuse treatment.” (response options = (a) 5%, (b) 10%, (c) 15%, or (d) 25% [correct answer]).

Unique items used for the MHC version of the test included: “Which of the following is NOT true about smoking and diabetes?” (response options = (a) there is no evidence that smoking causes type 2 diabetes [correct answer], (b) smoking leads to poor blood flow in the legs and feet, which can result in possible amputation, (c) people who smoke likely will need to use a higher dose of insulin, (d) smoking increases the likelihood of becoming blind for people with diabetes, and (e) all of the above are true); and “Which reason(s) contribute to patients not attempting to quit using tobacco products?” (response options = (a) lack of time and competing priorities during the patient visit, (b) very few patients have a desire to quit using tobacco, (c) many patients are not advised to quit by a healthcare professional, (d) all of the above, and (e) A & C only [correct answer]).

Unique items used for the SUTC version of the test included: “Which of the following are reasons for a high smoking rate among people with a substance use disorder?” (response options = (a) lack of access to healthcare, (b) targeted by tobacco marketing, (c) perceived benefit to stress and anxiety reduction, and (d) all of the above [correct answer]); and “Individuals with a (non-nicotine) substance abuse or mental health disorder represent about 25% of the United States population but consume about 40% of all cigarettes sold to adults.” (response options = true [correct answer] or false).

The knowledge test was administered to employees before and after the training. The total number of correct answers was tallied for pre- and post-test performance, respectively. The percent change in knowledge test score (i.e., knowledge gained) from pre- to post-training was calculated using the following formula: (post-test score—pre-test score)/pre-test score × 100. After the completion of training sessions at each participating center, the center’s program champion received summative feedback, including the number of employees trained and the percent knowledge gained overall at the center.

### 2.3. Data Analyses

Organizational and participant demographics were calculated using descriptive statistics. Comparisons by center type were achieved using Mann–Whitney tests due to the small number of participating centers.

For the primary analyses, generalized linear mixed models were used to compare pre-implementation knowledge and past-year training by center type. Next, linear mixed models were used to compare summative test scores representing knowledge gained by center type. This was calculated for both the 7 shared items version (possible range 0–7) and the 10-item (possible range 0–10) versions of the tests. All models accounted for nested data structure (providers within centers).

In all analytic comparisons, statistical significance was designated at *p* < 0.05, and analyses were conducted using SAS version 9.4.

## 3. Results

### 3.1. Center Demographics

The 16 participating centers (8 MHCs and 8 SUTCs) together served 98,703 unique patients yearly from 105 of Texas’ 254 counties (41.3%). Each participating center served at least one county designated as rural/partially rural; overall, 96 of the 105 (91.43%) counties served were rural or partially rural. The 96 included counties represent ~40% of all rural/partially rural counties in Texas. Of the 1572 employees at the centers, 1046 were healthcare providers (66.5%). There were no significant differences between MHCs and SUTCs on these or other demographic variables reported by their center’s leadership. See Table 1.

### 3.2. Pre-Implementation Tobacco Knowledge and Past-Year Training

Of the 1046 healthcare providers across 16 participating healthcare centers, 428 completed the pre-implementation survey, corresponding to a response rate of 40.92%.

Results indicated that 69.16% of responding providers knew that patients with behavioral health disorders were twice as likely than those in the general population to smoke cigarettes, 45.33% knew that they were more likely to die from their smoking-related illnesses than from their other SUD/s, 31.31% agreed that patients with SUDs want to quit and can quit smoking, and 60.28% knew that if they quit smoking they could experience concurrent improvements in other behavioral health symptoms. Overall, only 28.50% of employees reported receiving training on the hazards of smoking in the last year, and only 22.66% reported receiving training on smoking hazards and the benefits of quitting for individuals with SUDs. The only significant difference between center types was that MHC providers were less likely than SUTC providers to know that patients with behavioral health disorders like substance misuse were twice as likely as those in the general population to smoke cigarettes. See Table 2.

### 3.3. Pre- and Post-Training Tobacco Knowledge Tests

Overall, 324 providers completed the knowledge test before the training and 314 completed it after the session, corresponding to response rates of 30.98% and 30.02%, respectively, relative to the total number of providers in partnering centers. Slightly more providers completed the pre- than the post-tests due to unforeseen circumstances, such as job-related emergencies that prevented them from attending the entirety of the training.

According to both the shared/core 7-item summative score and the full 10-item summative score, there were significant gains in knowledge overall, as well as within the MHCs and the SUTCs, respectively. However, there were no significant differences between center types in the change between their pre-training test scores and their post-training test scores. See Table 3.

## 4. Discussion

This study examined tobacco-related knowledge and past-year training receipt among healthcare providers at SUTCs and MHCs, which together serve almost 100,000 patients annually who live in rural/partially rural counties and MUAs in Texas, USA. To our knowledge, this is the first study to directly compare provider knowledge and training on tobacco cessation across two settings where patients with SUDs receive healthcare, as well as to evaluate changes in knowledge following a brief educational intervention in each setting. Results indicated that knowledge was generally low at baseline, and training exposure in the past year was uncommon. Nonetheless, providers in both settings showed significant improvements in knowledge following the training, highlighting its potential as a practical first step in building capacity for tobacco cessation care.

Baseline knowledge varied across items but was generally low. Fewer than one-third of providers believed that patients with SUDs wanted to or could quit smoking, and fewer than half knew that these patients were more likely to die from smoking-related illnesses than their primary SUD. These gaps are critical because provider knowledge and attitudes significantly influence whether tobacco use care is delivered [36]. If providers do not recognize the risks of cigarette smoking or do not believe patients can quit, they may not initiate conversations about harm reduction strategies or cessation. Training efforts should therefore position conventional cigarette smoking as the most lethal form of tobacco use for patients in SUD treatment, and present research on the high proportion of smokers who are interested in quitting [37]. Additionally, training content should consist of actionable methods by which providers can assist their patients in gaining motivation for use reduction or a quit attempt, with the 5Rs intervention, for example [38].

Despite the differences in healthcare focus between MHCs and SUTCs, only one statistically significant difference in baseline knowledge emerged between center types: MHC providers were less likely than SUTC providers to know that people with behavioral health disorders are about twice as likely to smoke cigarettes as the general population. This might reflect greater familiarity among SUTC providers with their primary behavioral health population. Still, the practical implication is that neither group consistently understood key facts about tobacco use in this high-risk population. Additionally, past-year training exposure was uniformly low across both settings, with fewer than one in four providers reporting any education on the topic. Given the importance of training to improving provider knowledge and behavior, this represents a significant missed opportunity [20,21,22].

The low baseline knowledge and training rates likely reflect the broader challenges that centers in rural and MUAs face in addressing tobacco use. These include staffing shortages, limited time, a lack of provider confidence in delivering tobacco use care, limited awareness of cessation resources, and high rates of competing patient needs [14,29]. As a result, tobacco use becomes deprioritized in clinical encounters, despite its significant contribution to preventable morbidity and mortality in patients with SUDs [39]. Several models and frameworks highlight the importance of knowledge as foundational to behavior change [15,16,17,18]. In the application of Social Cognitive Theory, for example, improving provider knowledge can be seen as key to building confidence and, ultimately, increasing the likelihood of delivering tobacco cessation care. Given that low knowledge and training exposure were prevalent across both center types, these results reinforce that foundational education is still needed among providers routinely working with patients with SUDs. These systemic educational gaps help explain the study’s findings and highlight the importance of scalable training solutions, such as short, online modules or brief in-service training that can be easily implemented without placing additional strain on provider time or center resources. Although training alone is not sufficient to ensure delivery of tobacco cessation care, it is a necessary foundational step, especially when providers lack accurate information.

Encouragingly, the training delivered through this program was associated with significant gains in tobacco-related knowledge across both MHCs and SUTCs. Providers in both settings improved their performance on a 10-item test by approximately 42%, with no significant difference in knowledge gains by center type. That said, there was slightly greater knowledge gains among MHC providers (45.92%) compared to SUTC providers (37.42%). One possible explanation for this is that MHC providers had lower baseline knowledge, leaving more room for growth. It is also worth considering that the training content for MHCs may have been tailored in more relevant ways than the SUTC training was, which may have encouraged greater engagement with the content in these settings. Indeed, the implementation science literature suggests tailoring content to each center type’s specific needs may result in a better outcome [40]. Tailored content may help ensure the training feels relevant to providers’ day-to-day work and may increase engagement and application in practice. These were among the reasons we tailored the training to setting in this work. However, it remains unclear to what extent providers who completed the training perceived the work as relevant. Exploring this could be a valuable direction for future research.

Prior work conducted in urban or mixed urban/suburban healthcare centers in Texas has also supported significant knowledge gains from a brief tobacco training. For example, in a study of urban opioid treatment centers, there were similar or slightly lower knowledge gains following a comparable (but setting-tailored) training [22]. Other work conducted in a range of SUTCs found baseline knowledge scores between 4.6 and 6.2 with gains reaching the 8-point range post-training, as was the case in this work [21]. Thus, knowledge gain levels seen herein, averaging over 40% improvement, suggest that even in rural or resource-limited settings, providers respond well to structured, targeted education, bolstering the generalizability of this approach.

Although MHCs and SUTCs differ in structure and mission, the lack of statistically significant differences in their demographic characteristics, including provider count, patient volume, and service reach, suggests some similarities in context for implementation. However, noted are potential differences in the staff to patient ratio between center types and a wide variability in center size, with some employing only a few providers and others over 300. These differences imply that future training initiatives may need to vary in format and intensity to meet the logistical realities of different-sized centers.

These findings offer helpful guidance for key stakeholders. For funders, the results demonstrate the value of low-cost, scalable educational interventions in rural and underserved regions, and support the need for implementation science programs that can help to usher evidence-based tobacco interventions into real-world clinical settings. For providers and organizational leaders, the data emphasize the need to make tobacco training more routine, ideally incorporated into new employee onboarding and annual training, to ensure providers stay current with new products and treatment strategies. For policymakers and rural health advocates, this work points to the importance of expanding access to evidence-based tobacco education, especially in areas with a high tobacco-related disease burden. Finally, given the success of this brief training model, building provider capacity to deliver evidence-based tobacco treatment has the potential to reduce the burden of smoking-related disease among some of Texas’s most at-risk communities.

### Study Limitations, Strengths, and Future Directions

Limitations of this study include using a convenience sample of healthcare centers that opted to participate in a tobacco-free workplace program. Providers at these centers may have been more likely to respond to recruitment efforts because they worked in organizations already interested in building their tobacco cessation capacity, which could introduce selection bias. In that case, the findings presented here may reflect more favorable knowledge or training outcomes than observed in centers less motivated or equipped to address tobacco use. Conversely, it is possible that centers not participating in the program perceived themselves as already meeting standards for tobacco care delivery or had limited capacity to participate as the result of competing demands, regardless of potential interest and need. Thus, the data presented may under- or over-estimate tobacco control capacity in Texas’ rural and underserved areas. Future work should examine reasons for center non-participation to better inform next steps toward capacity building in the state.

Moreover, it is important to also note that data may not fully represent all providers in respective participating centers. Although leadership supported their full participation, response rates for the survey and the training knowledge tests suggested only 30–41% of the providers participated. Reasons for non-participation in training or in data collection are not fully known, but at least some were attributable to competing job demands. However, the non-participation estimate was based on data gathered at a static point in time (i.e., the number of providers at the partnering organizations prior to program implementation/training), whereas the procedures described and data collected herein occurred over time; staffing numbers could have varied accordingly in unknown ways, as is common in real-world implementation projects. Regardless, future studies in this area should consider other ways of incentivizing participation in training activities and data collection efforts to engage a greater proportion of providers in these activities. This speaks again to the value of scalable training solutions that can be delivered without straining provider time or resources.

Another limitation is that all data from providers were self-reported. Their responses regarding tobacco-related knowledge, past-year training, and post-training knowledge gains may not perfectly reflect their true beliefs, exposure, or understanding. Some responses may have been influenced by social desirability bias, while others could have been affected by recall limitations. Surveys were anonymous to help reduce potential effects of social desirability, and respondents were informed that individual results would not be shared with their center’s leadership. Still, self-report measures carry inherent limitations. In addition, the study focused narrowly on knowledge as the primary outcome. It did not capture other meaningful constructs such as provider self-efficacy, motivation, or actual behavior change following training. Additionally, knowledge may fade over time; the present study measured knowledge gain immediately following training, and it is unknown how much information may have been retained over time. Finally, our assessment of knowledge gain focused heavily on items about cigarette smoking because of the strength of the research on conventional cigarette use and because it is the most common form of tobacco use. As such, knowledge about the intricacies of other, non-cigarette forms of tobacco use reviewed in the training is unknown and requires further research.

The lack of provider demographic data is another gap in the current work. Information such as clinical discipline, years of experience, and educational background could have helped contextualize knowledge levels and training needs. Moreover, because assessment procedures were de-identified, we did not have the ability to link individual-level data from the surveys, the pre-training, or the post-training knowledge tests. Although the chosen procedures were to minimize respondent concern and encourage truthfulness, future studies could collect identifiable data to link survey responses with training outcomes for a more in-depth examination. Similarly, information about the patient populations served (e.g., smoking prevalence, insurance coverage) would add value in understanding how these factors shape the delivery of tobacco care in different settings. However, in settings with limited tobacco cessation knowledge (often accompanied by inconsistent screening), pre-implementation estimates of smoking prevalence may be substantially underestimated, making accurate assessment challenging.

Although prior work has supported the potential impact of training on provider behavior change [20,21,22], we cannot determine whether improvements in knowledge will lead to changes in clinical behavior or improved patient outcomes with the data reported herein for this ongoing study. Future work should use longitudinal designs to evaluate whether increased knowledge from training leads to sustained changes in provider practices and ultimately improves tobacco cessation rates among patients with SUDs.

The study’s design also offers several strengths worth noting. The focus on healthcare centers serving rural and MUAs, which are often underrepresented in research, provides valuable insight into provider preparation to address patients’ tobacco use in high-need regions. The collaboration with real-world healthcare centers adds to the ecological validity of the study. Additionally, the inclusion of multiple centers across a wide geographic area enhances the breadth of the findings and captures diverse clinical contexts within Texas, USA. Still, although the study focused on healthcare centers across a wide geographic area in Texas, home to a large rural population and hundreds of MUAs, the findings may not generalize to rural or underserved areas in other states (or countries) where healthcare systems, state/national tobacco-free policies, patient demographics, and provider training may differ. Expanding this work to include additional states, regions, or countries would help build a more representative understanding of provider knowledge and tobacco care practices in these settings. Finally, it is important to note that the designation of areas as rural/partially rural or medically underserved may differ based on the source of the data and time of data collection. Consequently, the designations used in the present work may not generalize to other sources and/or other timeframes.

## 5. Conclusions

Our findings suggest that, across both SUTCs and MHCs serving rural/partially rural counties and/or MUAs in Texas, provider knowledge about tobacco use in patients with SUDs was limited, and past-year training on smoking cessation was not very common. Although minimal significant differences were observed between center types in overall knowledge levels or training exposure, the variability in baseline scores and post-training gains suggests room for tailored educational efforts across settings. Brief training, including on the 5A’s and 5R’s and tailored to the provider audience’s typical scope of practice, significantly improved provider knowledge in both MHCs and SUTCs, indicating that such education may be a practical entry point for building clinical capacity. Given that tobacco use continues to drive health inequities in these populations, annual or onboarding training on both cigarettes and non-combustible tobacco products may help ensure providers remain equipped to address all forms of tobacco dependence. Training interventions should be adapted to each center type, accounting for staffing differences and service focus. Continued efforts to implement and evaluate tobacco-free workplace programs that include structured provider education are warranted, especially in settings with lower baseline knowledge or greater reported barriers to care. Future research is needed to assess the generalizability of these findings outside of Texas and to determine whether knowledge improvements translate into long-term changes in provider behavior and patient outcomes.

## Figures and Tables

**Table 1:** Pre-implementation Characteristics of Medical Healthcare Centers (MHCs) and Substance Use Treatment Centers (SUTCs) Serving Rural and/or Medically Underserved Areas in Texas, USA that Agreed to Participate in a Comprehensive Tobacco-free Workplace Program (N = 16).

Select Center Characteristics	All Centers (N = 16)		MHCs (n = 8)		SUTCs (n = 8)		Statistic & *p*-Value	
Number of…	Mean (SD)	Sum (Range)	Mean (SD)	Sum (Range)	Mean (SD)	Sum (Range)		
counties served	8.31 (11.11)	105 (1, 40)	3.50 (2.33)	25 (1, 7)	13.13 (14.36)	93 (1, 40)	80.5	0.2000
rural or partially rural counties served	6.94 (9.96)	96 (1, 33)	2.50 (1.77)	20 (1, 6)	11.38 (12.83)	85 (1, 33)	80.0	0.2174
counties with medically underserved areas served	5.75 (7.88)	72 (0, 29)	2.38 (1.51)	16 (0, 5)	9.13 (10.23)	64 (1, 29)	81.0	0.1915
unique patients seen yearly	6168.94 (11,348.24)	98,703 (40, 46,000)	5607.25 (4770.92)	44,858 (1000, 12,139)	6730.63 (15,889.61)	53,845 (40, 46,000)	56.0	0.2328
total patient visits yearly	21,271.50 (30,512.10)	340,344 (175, 98,000)	16,968.75 (12,418.62)	135,750 (1750, 40,000)	25,574.25 (42,407.97)	204,594 (175, 98,000)	56.5	0.2460
employees (inclusive of healthcare providers)	98.25 (149.52)	1572 (5, 605)	77.25 (70.54)	618 (12, 215)	119.25 (204.75)	954 (5, 605)	63.0	0.6454
healthcare providers	65.38 (90.92)	1046 (5, 350)	53.00 (56.59)	424 (9, 174)	77.75 (119.01)	622 (5, 350)	66.0	0.8785

Note. Mann–Whitney tests were conducted to assess differences by center type. Rural counties are defined as those in which all census tracts are rural areas and partially rural counties are those where at least one (but not all) census tract is a rural area [8,9]. Counties with medically underserved areas have at least one geographic area with a shortage of primary care health services or with a shortage of primary care health services for specific populations [10,11,12]. The counties served, rural or partially rural counties served, and counties served with medically underserved areas in the table represent unique counts; stated differently, if more than 1 center served the same county, the county was only counted once in the table. It is important to note that rural/partially rural and medically underserved areas change over time; data reported above were gathered from the Texas State Office of Rural Health, Health Resources and Services Administration in November 2024 [9,12]. SD = standard deviation.

**Table 2:** Pre-Implementation Employee Knowledge on Smoking Cessation Care and its Benefits for Patients with Behavioral Health Disorders and Past-Year Tobacco Training by Center Type During the Implementation of a Comprehensive Tobacco-Free Workplace Program in Texas, USA (N = 428).

	All (N = 428)	MHCs (n = 203)	SUTCs (n = 225)	*p*-Value
	% Yes (n)			
Knowledge Statements				
People with mental health and/or non-nicotine substance use disorders…				
are ~2× as likely as the general population to smoke cigarettes	69.16 (296)	64.53 (131)	73.33 (165)	0.0492
are more likely to die from smoking-related illnesses than from their mental and/or non-nicotine substance use disorder	45.33 (194)	48.77 (99)	42.22 (95)	0.1751
who smoke want to quit smoking and are able to quit smoking	31.31 (134)	31.03 (63)	31.56 (71)	0.6422
who quit smoking may experience concurrent improvements in some mental health symptoms and/or reductions in non-nicotine substance use	60.28 (258)	61.08 (124)	59.56 (134)	0.7477
Education Receipt				
Past-year receipt of education on the hazards of smoking	28.50 (122)	25.12 (51)	31.56 (71)	0.1017
Past-year receipt of education on the hazards of smoking and benefits of quitting that are specific to individuals with substance use disorders	22.66 (97)	23.65 (48)	21.78 (49)	0.6080

Note. Generalized linear mixed models were used to assess differences between center types, accounting for the clustering of healthcare providers within center. MHC = medical healthcare center; SUTC = substance use treatment center.

**Table 3:** Knowledge Gain from Pre-to Post-training Provided During the Implementation of a Comprehensive Tobacco-Free Workplace Program by Center Type in Texas, USA (N = 16 centers, 324 providers).

		All Centers (N = 16)				MHCs (n = 8)				SUTCs (n = 8)			
		Pre	Post	*p*-Value	% Change	Pre	Post	*p*-Value	% Change	Pre	Post	*p*-Value	% Change
	N (of Providers)	324	314			176	173			148	141		
	Possible Score Range	Mean (SD)				Mean (SD)				Mean (SD)			
7 (Shared) Item Total	0–7	3.92 (1.58)	5.89 (1.24)	<0.0001	50.23	4.01 (1.57)	5.95 (1.24)	<0.0001	48.63	3.82 (1.58)	5.81 (1.23)	<0.0001	52.15
10 Item Total	0–10	5.84 (1.96)	8.27 (1.59)	<0.0001	41.69	5.49 (1.98)	8.02 (1.72)	<0.0001	45.92	6.25 (1.85)	8.59 (1.37)	<0.0001	37.42

Note. Linear mixed models were used, accounting for nesting of healthcare providers within center. The moderation effect of center type was not significant in the relationship between the center type and knowledge test scores (*p* = 0.8518 for shared items; *p* = 0.4975 for 10-item total score). MHC = medical healthcare center; SUTC = substance use treatment center; SD = standard deviation.

## Data Availability

The data presented in this study are available on request from the corresponding author. The data are not publicly available because the study is ongoing.

## Abbreviations

The following abbreviations are used in this manuscript:

USA	United States of America
SUD	Substance use disorder
MUA	Medically underserved area
MHC	Medical healthcare center
SUTC	Substance use treatment center
